# Correction: Shen, T. et al. High-Precision and Low-Cost Wireless 16-Channel Measurement System for Malachite Green Detection. *Micromachines*, 2018, *9*, 646

**DOI:** 10.3390/mi10020142

**Published:** 2019-02-21

**Authors:** Tong Shen, Tong Zhou, Ying Wan, Yan Su

**Affiliations:** School of Mechanical Engineering, Nanjing University of Science and Technology, Nanjing 210094, China; shentong@njust.edu.cn (T.S.); yingwan@njust.edu.cn (Y.W.); yansu@njust.edu.cn (Y.S.)

In the published paper [1], there is an error in Figure 7. The sentence “We found that the amperometric signal was logarithmically related to the sample concentration in a range from 1 μg/L to 1 mg/L, which spanning a response region of at least 3 orders of magnitude, as shown in Figure 7d.” should read as “We found that the amperometric signal was logarithmically related to the sample concentration in a range from 1 μg/L to 1 mg/L, which spanning a response region of at least 3 orders of magnitude, as shown in Figure 7e.” Figure 7 should be corrected as follows:

The changes do not affect the scientific results. We apologize for any inconvenience caused to the readers by these errors. The manuscript will be updated, and the original will remain online on the webpage for the article including a reference to this Correction.

## Figures and Tables

**Figure 7:**
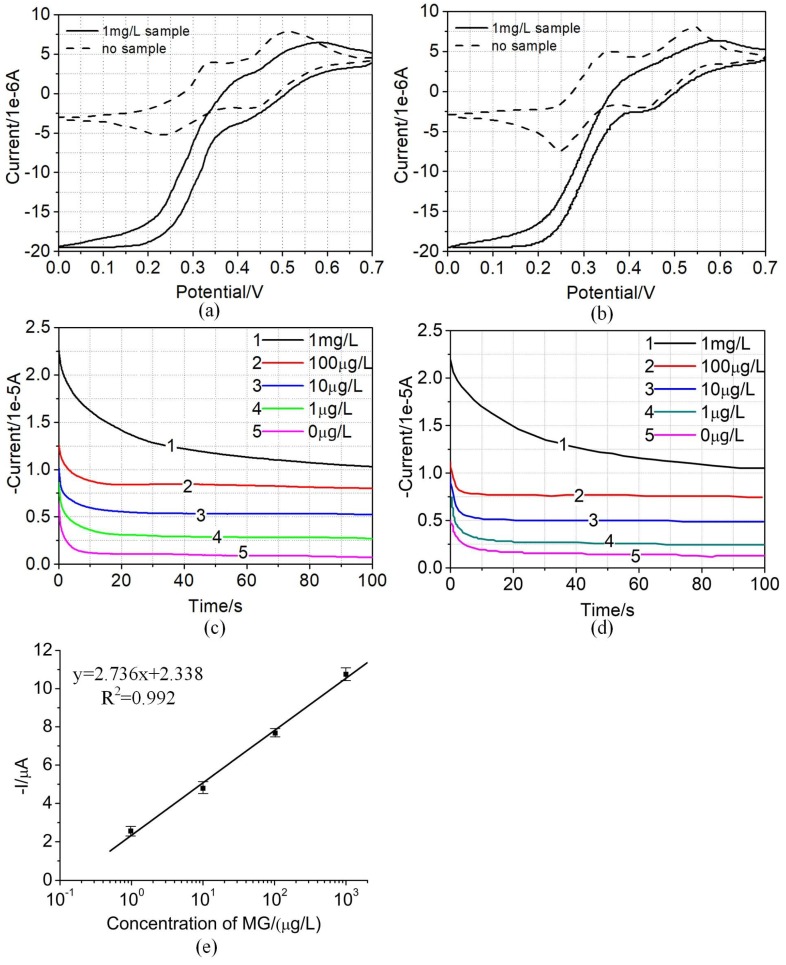
Detection performance of MG. (**a**) Cyclic voltammograms for no target (dashed line) and 1 mg/L target (solid line) obtained from commercial electrochemical instrument. (**b**) Cyclic voltammograms for no target (dashed line) and 1 mg/L target (solid line) obtained from the hand-held electrochemical instrument in this system. (**c**) Amperometric curves of samples with different concentrations (1 mg/L, 100 μg/L, 10 μg/L, 1 μg/L, and 0 μg/L) in TMB substrate solution obtained from commercial electrochemical instrument. (**d**) Amperometric curves of samples with different concentrations (1 mg/L, 100 μg/L, 10 μg/L, 1 μg/L, and 0 μg/L) in TMB substrate solution obtained from the hand-held electrochemical instrument in this system. (**e**) A calibration plot of the amperometric current and the log concentration of target. Data were collected from at least three independent experiments.
